# Imported hepatopulmonary echinococcosis: first report of *Echinococcus granulosus* sensu stricto (G1) in Bolivia

**DOI:** 10.1590/0037-8682-0046-2018

**Published:** 2020-01-27

**Authors:** Daniel Jarovsky, Clarissa Rodrigues da Silva Brito, Danieli Urach Monteiro, Maria Isabel de Azevedo, Sônia de Avila Botton, Marcelo Jenné Mimica, Mariana Volpe Arnoni, Marco Aurélio Palazzi Sáfadi, Eitan Naaman Berezin, Humberto Salgado, Flavia Jacqueline Almeida, Mário Luiz de la Rue

**Affiliations:** 1 Santa Casa de São Paulo, Serviço de Infectologia Pediátrica, São Paulo, SP, Brasil.; 2 Universidade Federal de Santa Maria, Departamento de Microbiologia e Parasitologia, Santa Maria, RS, Brasil.; 3 Universidade Federal de Minas Gerais, Departamento de Medicina Veterinária Preventiva, Belo Horizonte, MG, Brasil.; 4 Universidade Federal de Santa Maria, Departamento de Medicina Veterinária Preventiva, Santa Maria, RS, Brasil.; 5 Santa Casa de São Paulo, Serviço de Controle de Infecção Hospitalar, São Paulo, SP, Brasil.; 6 Santa Casa de São Paulo, Serviço de Cirurgia Pediátrica, São Paulo, SP, Brasil.

**Keywords:** Echinococcus granulosus sensu stricto, Hepatopulmonary hydatidosis, Echinococcosis

## Abstract

Hepatopulmonary hydatidosis in young children is a rare and atypical presentation of *Echinococcus granulosus* infection. We report the first case of cystic echinococcosis caused by a microvariant of *E. granulosus sensu stricto*. Chemotherapy and systemic corticoids were administered before curative surgery was performed. Recurrence was not observed for more than 24 months of follow-up.

## INTRODUCTION

**FIGURE 1:**
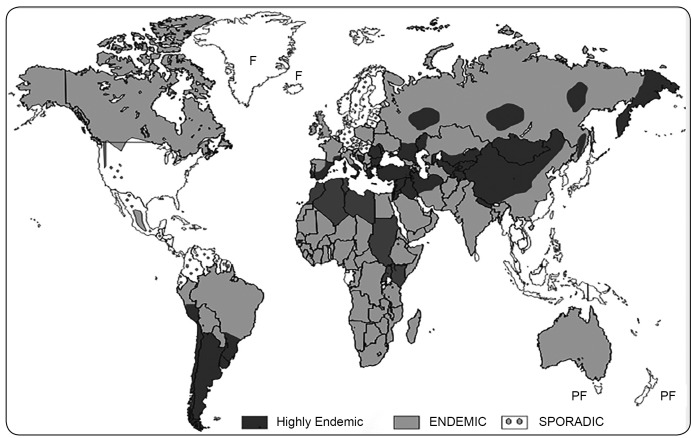
Estimated worldwide distribution and geographical endemicity of the zoonotic strains of *Echinococcus granulosus* (adapted from reference 6). **F:** free; **PF:** provisionally free.

Cystic echinococcosis (CE) or hydatidosis is a globally neglected zoonotic disease and is highly ranked in the list of food-borne parasites distributed worldwide^1^. Infection is caused by tapeworms belonging to the genus *Echinococcus* (family Taeniidae) and leads to the development of solitary or multiple slow-growing cysts in the liver and lungs in 86% to 97.6% of cases. It is endemic in several parts of the world, particularly in South America, with under-reported global estimates of more than one million human CE cases yearly^2^ (Figure 1).

The *Echinococcus* species is indistinguishable morphologically, with considerable genetic and phenotypic variation. *E. granulosus* complex or *sensu lato* is a complex of ten defined species/strains, identified based on morphology, host specificity, and mitochondrial DNA sequences^2^. Epidemiological data on these strains are lacking in many regions of the world. We describe a rare case of combined CE in a young Bolivian child and the first human identification of a new genetic sequence of *E. granulosus sensu stricto* (G1).

## CASE REPORT

A three-year-old girl was admitted to the Santa Casa de São Paulo Hospital. She complained of abdominal pain and increased abdominal volume for approximately one year. Her symptoms worsened five days before admission. No fever, vomiting, diarrhea, or respiratory symptoms were reported. She was born in a rural area in La Paz, Bolivia, but she had been living in São Paulo, Brazil, for the last three months. She was previously healthy. She was frequently exposed to sheep in a farm and came into direct contact with domestic dogs while residing at her birthplace.

The child was mildly pale and tachypneic (respiratory rate of 27 breaths/minute), but otherwise well. Her abdomen was bulky and soft, and she felt no pain in her abdomen during examination. Two masses with smooth edges were palpable in both hypochondriac regions, ±6 cm and ±2 cm from the right and left rib margins, respectively. Respiratory murmurs were slightly diminished during the right lung auscultation. The additional parameters assessed during clinical examination and laboratory evaluation were unremarkable.

**FIGURE 2:**
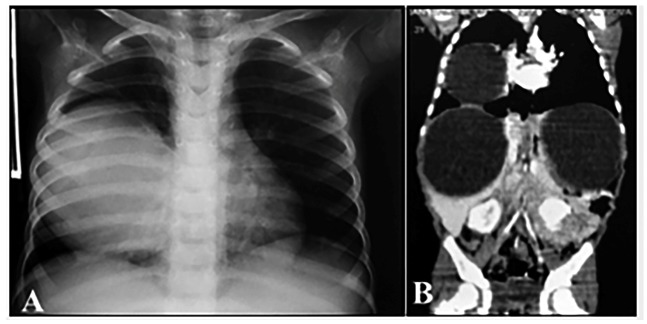
Anteroposterior chest radiograph **(A)** and coronal thoracoabdominal computed tomography scan **(B)** revealing large combined cystic masses in both the thoracic and abdominal cavities.

Chest radiography and computed tomography (CT) revealed a round and well-defined cystic mass measuring 10×7.4×6.3 cm (±465 cm^3^) partially occupying the middle and lower thirds of the right hemithorax and laminar pleural effusion on the same side (Figure 2A). Abdominal CT scan revealed bilateral sub-diaphragmatic, hypodense, and homogeneous cystic formations, measuring approximately ±9.3×8.5 cm on the right side and ±9.8×7.5 cm on the left side, which displaced the abdominal organs (Figure 2B).

CE was highly suggestive. Therefore, albendazole chemotherapy (15 mg/kg/day) was administered for five days before elective thoracoscopy and laparotomy to reduce the rate of secondary echinococcosis (i.e., the release of protoscoleces following a spontaneous or trauma-induced cyst rupture) during the surgical procedure. A two-stage surgical excision was performed via posterolateral thoracotomy and laparotomy completely removed all cystic masses uneventfully. Systemic corticoids were administered before surgery to prevent anaphylactic shock.

Serum antibody detection tests were not performed. Total DNA was extracted from the protoscoleces suspension in the hydatid cyst, and molecular analysis was performed using the cytochrome c oxidase subunit 1 (*cox1*) mitochondrial gene primer. The forward and reverse primers used were *coxI*.For/*coxI*.Rev (366 bp), as described by Bowles et al^3^. Comparative DNA sequence analyses were performed using the BLAST program (Basic Local Alignment Search Tool) and Staden Package Gap4. Gene sequences were compared to those available in the GenBank database (https://www.ncbi.nlm.nih.gov/genbank). This revealed a new variant (KU168961) that showed 100% homology to other *E. granulosus sensu stricto* (G1) sequences (unpublished data, available at http://www.ncbi.nlm.nih.gov/nuccore/KU168961.1). 

The patient was discharged after 15 days of treatment, with resolved abdominal pain and tachypnea and no residual sequelae. Complete cure was achieved after three months of treatment. No recurrence was observed for more than 24 months of follow-up.

## DISCUSSION

The *E. granulosus* complex and its genetic variants were introduced into South America via domestic animals imported primarily from Europe and some African regions^4^. Due to its high infectivity in humans, *E. granulosus sensu stricto* is an important cause of CE in endemic countries. Some sources estimate that the true incidence of the disease could be up to 100 times greater than reported^5^.

The biological life cycle of these parasites involves two mammalian hosts: a definitive host, wherein the adult cestode inhabits the small intestine of a carnivore (usually wild or domestic canids), and an intermediate host (wild or livestock mammals, mainly sheep, swine, cattle, camelids, and goats), wherein tissue-invading larval stages (metacestodes) develop in internal organs following oral intake of tapeworm eggs released by a carnivore. Humans are accidental intermediate hosts and are infected through the handling of infected definitive hosts, egg-containing feces, or egg-contaminated plants or soil followed by direct hand-to-mouth transfer^6^. 

The *E. granulosus* complex and *E. multilocularis* are the most important members of the genus, due to their public health importance and geographical distribution^7^. *E. granulosus* can cause liver (70%) and lung (20%) unilocular hydatid cysts in humans, while infection with *E. multilocularis* results in alveolar echinococcosis: a series of small, interconnected cysts virtually restricted to the liver.

Currently, the *E. granulosus* complex is composed of *E. granulosus sensu stricto* (G1, G2, and G3 genotypes), *E. equinus* (G4 genotype), *E. ortleppi* (G5 genotype), *E. canadensis* (G6, G7, G8, and G10 genotypes), and *E. felidis* (G9, “lion strain”) [Supplementary material: Table 1 and Reference 8]. Genotype 1 is associated with common sheep and is responsible for ±90% of all human episodes worldwide^8^. 

Among the South American countries, the disease is endemic in Argentina, southern Brazil, Uruguay, Chile, and the mountainous regions of Peru and Bolivia. Almost 30,000 new cases of CE in humans were registered by these countries from 2009 to 2014; 70% of these cases were reported in Peru, while only 0.3% of these cases were reported in Brazil^9^.

In Brazil, *E. granulosus* is endemic to the Rio Grande do Sul and Santa Catarina provinces. Santa Casa Hospital is situated in downtown São Paulo, and no autochthonous cases were reported in this large metropolitan area. In this study, we describe a rare imported case of combined pulmonary and liver hydatidosis in a young child. The majority of the reported cases involved children who were more than 4-years-old. Additionally, in 5-10% of cases, two or more organs are simultaneously affected^10^. Surgery is the first choice of treatment for multiple and large cysts, and therapy with anti-parasitic drugs is indicated as an adjuvant to surgery to decrease the number of relapses and hydatid cyst size before the surgical procedure. Pharmacological management relies on benzimidazole compounds (albendazole or mebendazole), which may be combined with praziquantel or nitazoxanide.

The lack of *E. granulosus* molecular information from Bolivia is an additional limitation to the epidemiologic characterization of the disease in the country. This makes our unique finding particularly relevant. Additionally, to the best of our knowledge, this is one of the first cases of CE in human caused by genotype 1 in Bolivia and probably the first to be documented in a child^11^ ^,^ ^12^. 

In conclusion, CE should be considered as a major diagnosis when there is the presence of large cystic masses and a highly suggestive epidemiology (pastoral and poor rural communities in a highly endemic country or region where people raise livestock and are in close contact with dogs). Similar to other neglected conditions, CE is also under-reported.

## References

[1] Budke CM, Deplazes P, Torgerson PR (2006). Global socioeconomic impact of cystic
echinococcosis. Emerg Infect Dis.

[2] Kinkar L, Laurimäe T, Acosta-Jamett G, Andresiuk V, Balkaya I, Casulli A (2018). Global phylogeography and genetic diversity of the zoonotic
tapeworm Echinococcus granulosus sensu stricto genotype G1. Int J Parasitol.

[3] Bowles J, Blair D, McManus DP (1992). Genetic variants within the genus Echinococcus identified by
mitochondrial DNA sequencing. Mol Biochem Parasitol.

[4] Rodero Franganillo A, Delgado JV, Rodero Serrano E (1992). Primitive andalusian livestock and their implications in the
discovery of America. Archivos de Zootecnia.

[5] Larrieu EJ, Frider B (2001). Human cystic echinococcosis: contributions to the natural history
of the disease. Ann Trop Med Parasitol.

[6] Eckert J, Deplazes P (2004). Biological, epidemiological, and clinical aspects of
echinococcosis, a zoonosis of increasing concern. Clin Microbiol Rev.

[7] Grosso G, Gruttadauria S, Biondi A, Marventano S, Mistretta A (2012). Worldwide epidemiology of liver hydatidosis including the
Mediterranean area. World J Gastroenterol.

[8] Alvarez Rojas CA, Romig T, Lightowlers MW (2014). Echinococcus granulosus sensu lato genotypes infecting
humans--review of current knowledge. Int J Parasitol.

[9] Pavletic CF, Larrieu E, Guarnera EA, Casas N, Irabedra P, Ferreira C (2017). Cystic echinococcosis in South America: a call for
action. Rev Panam Salud Publica.

[10] Budke CM, Carabin H, Ndimubanzi PC, Nguyen H, Rainwater E, Dickey M (2013). A Systematic Review of the Literature on Cystic Echinococcosis
Frequency Worldwide and Its Associated Clinical
Manifestations. Am J Trop Med Hyg.

[11] Schantz PM (1972). Hydatidosis: scope of the problem and prospects of its
control. Bol Oficina Sanit Panam.

[12] Kamenetzky L, Gutierrez AM, Canova SG, Haag KL, Guarnera EA, Parra A (2002). Several strains of Echinococcus granulosus infect livestock and
humans in Argentina. Infect Genet Evol J Mol Epidemiol Evol Genet Infect Dis.

