# Supplementary material for: Imported hepatopulmonary echinococcosis: first report of Echinococcus granulosus sensu stricto (G1) in Bolivia
Source: Rev Soc Bras Med Trop. 2020 Jan 27;53:e20180046. doi: 10.1590/0037-8682-0046-2018 (PMC7083390; doi:10.1590/0037-8682-0046-2018)
Supplement: Supplementary file 1 [file 1678-9849-rsbmt-53-e20180046-suppl1.pdf]

TABLE 1: Current taxonomy for the genus *Echinococcus* (adapted from references 2 and 3, Laurimäe et al. [Parasitology. 2018;145(14):1929–37], Yanagida et al. [Int J Parasitol. 2017;47(14):971–9], Alvarez Rojas et al. [Int J Parasitol. 2014;44(1):9–18], McManus et al. [Parasitology. 2003;127 Suppl:S37-51] and Nakao et al. [Int J Parasitol. 2013;43(12–13):1017–29]).

Species	Genotype, strain	Distribution	Definitive hosts	Intermediate hosts	Human infections
E. granulosus senso stricto	G1 - Common sheep strain	Worldwide	**Dog**, fox, dingo, jackal, hyena	**Sheep**, cattle, pig, camel, goat, macropods	Commonest
E. granulosus senso stricto	G2 - Tasmanian sheep strain	Tasmania, Argentina	**Dog**, fox	**Sheep**, cattle (?)	Commonest
E. granulosus senso stricto	G3 - Buffalo strain	Asia	**Dog**, fox (?)	**Bovine** (water buffalo, cattle?), sheep	Commonest
Echinococcus equinus	G4 - Horse strain	Worldwide	**Dog**	Equines (**horse**, donkey, zebra)	Unknown
Echinococcus ortleppi	G5 - cattle strain	Europe, South Africa, India, Nepal, Sri Lanka, Russia, South America (?)	**Dog**	**Bovine** (buffalo, cattle), sheep, goat, pig	Less common
Echinococcus canadensis	G6 - Camel strain	Middle East, Iran, Africa, China, Nepal, Argentina	**Dog**	**Camelids**, goat, cattle	Common
Echinococcus canadensis	G7 - Pig strain	Poland, Slovakia, Ukraine, Russia, Argentina	**Dog**	Pig	Common
Echinococcus canadensis	G8 - Cervid strain	Holarctic zone (North America, Northern Eurasia)	**Wolf**, dog	American cervids (moose, wapiti, muskox)	Uncommon
Echinococcus felidis	G9 - Lion strain	Africa	Lion, spotted hyena	Warthog, zebra, wildebeest, bush pig, buffalo, various antelope species, giraffe (?), hippopotamus (?)	Unknown
Echinococcus canadensis	G10 - Cervid strain	Holarctic zone (North America, Northern Eurasia)	**Wolf**, dog	Fennoscandian cervids (reindeer, wapiti, moose)	Uncommon
E. multilocularis	-	Holarctic zone (North America, Northern Eurasia)	Fox, dog	Voles	Common
E. shiquicus	-	Tibetan plateau	Tibetan fox	Tibetan fox, plateau pika	Unknown
E. oligarthra	-	Neotropical (South America)	Wild felids (leopard, puma)	Agouti	Uncommon
E. vogeli	-	Neotropical (South America)	bush dog	Paca	Uncommon
